# Supplementary material for: Advancing artificial intelligence applicability in endoscopy through source-agnostic camera signal extraction from endoscopic images
Source: PLoS One. 2025 Jun 11;20(6):e0325987. doi: 10.1371/journal.pone.0325987 (PMC12157078; doi:10.1371/journal.pone.0325987)
Supplement: S1 Table — The EPIC dataset contains 267 images stored using 9 different endoscopic processors, several endoscopes and different recording aspect ratio settings. ERCP: Endoscopic Retrograde Cholangiopancreatography, EPIC: Endoscopic Processor Image Collection. (DOCX) [file pone.0325987.s001.docx]

**S1 Table:** **Image data from endoscopic processors included in the EPIC dataset.** The EPIC dataset contains 267 images stored using 9 different endoscopic processors, several endoscopes and different recording aspect ratio settings. ERCP: Endoscopic Retrograde Cholangiopancreatography, EPIC: Endoscopic Processor Image Collection

| **Processor** | **Endoscope** | **Manufacturer** | **Intervention** | **4:3 Available** | **5:4 Available** |
| --- | --- | --- | --- | --- | --- |
| Olympus (Olympus Europa SE & Co. KG, Hamburg, Germany) | | | | | |
| CV-180 | CF-Q145I | Olympus | Colonoscopy | Yes | Yes |
|  | GIF-Q145 | Olympus | Gastroscopy | Yes | Yes |
|  | GIF-XQ140 | Olympus | Gastroscopy | Yes | Yes |
| CV-190 | SpyGlass | Boston Scientific | Cholangioscopy | No | No |
|  | CF-H180AL | Olympus | Colonoscopy | Yes | Yes |
|  | CF-HQ190L | Olympus | Colonoscopy | Yes | Yes |
|  | CF-Q160AL | Olympus | Colonoscopy | Yes | Yes |
|  | CF-HQ190L | Olympus | Colonoscopy | No | No |
|  | GF-UE190 | Olympus | Gastroscopy | No | No |
|  | GF-UTC180 | Olympus | Gastroscopy | No | No |
|  | GIF-1T140 | Olympus | Gastroscopy | No | No |
|  | GIF-1T190 | Olympus | Gastroscopy | No | No |
|  | GIF 2T160 | Olympus | Gastroscopy | No | No |
|  | GIF-H180 | Olympus | Gastroscopy | No | No |
|  | GIF-H185 | Olympus | Gastroscopy | No | No |
|  | GIF-H190N | Olympus | Gastroscopy | No | No |
|  | GIF-HQ190 | Olympus | Gastroscopy | No | No |
|  | GIF-XP190N | Olympus | Gastroscopy | No | No |
|  | GIF-XTQ160 | Olympus | Gastroscopy | No | No |
|  | PCF-H140L | Olympus | Colonoscopy | Yes | Yes |
|  | PCF-H180AL | Olympus | Colonoscopy | No | No |
|  | PSF-1 | Olympus | Enteroscopy | No | No |
|  | SIF-Q180 | Olympus | Enteroscopy | No | No |
|  | TJF-140R | Olympus | ERCP | No | No |
|  | TJF-160R | Olympus | ERCP | No | No |
|  | TJF-160VR | Olympus | ERCP | No | No |
|  | CHF-V | Olympus | Choledoscopy | No | No |
| CV-1500 | CF-EZ1500DL | Olympus | Colonoscopy | No | No |
|  | CF-HQ190 | Olympus | Colonoscopy | No | No |
|  | GIF-EZ1500 | Olympus | Gastroscopy | No | No |
|  | GIF-HQ190 | Olympus | Gastroscopy | No | No |
| Fujifilm (FUJIFILM Europe GmbH, Düsseldorf, Germany) | | | | | |
| VP-4450HD | EC-590WL | Fujifilm | Colonoscopy | No | No |
|  | EG-530CT | Fujifilm | Gastroscopy | No | No |
| VP-7000 | EG-760CT | Fujifilm | Gastroscopy | No | No |
|  | EC-760R VI | Fujifilm | Colonoscopy | No | No |
| Pentax (PENTAX Europe GmbH, Hamburg, Germany) | | | | | |
| EPK i | EC-38-iF10F2 | Pentax | Colonoscopy | No | No |
|  | EC-3890Li | Pentax | Colonoscopy | No | No |
|  | ED34-i10T | Pentax | ERCP | No | No |
|  | EG29-i10 | Pentax | Gastroscopy | No | No |
|  | EG38-J10UT | Pentax | Gastroscopy | No | No |
|  | EG-2990i | Pentax | Gastroscopy | No | No |
|  | G-EYE34-i10L | Pentax | ERCP | No | No |
| EPK i7000 | EC38-i10F2 | Pentax | Colonoscopy | No | No |
|  | EC-3890Li | Pentax | Colonoscopy | No | No |
|  | ED34-i10T | Pentax | ERCP | No | No |
|  | EG29-i10 | Pentax | Gastroscopy | No | No |
|  | EG38-J10UT | Pentax | Gastroscopy | No | No |
|  | EG-2990i | Pentax | Gastroscopy | No | No |
|  | G-EYE34-10L | Pentax | ERCP | No | No |
| EPK i 7010 | EC-3890LI | Pentax | Colonoscopy | No | No |
|  | EC-i10F2 | Pentax | Colonoscopy | No | No |
|  | ED34-i10T | Pentax | ERCP | No | No |
|  | EG29-i10 | Pentax | Gastroscopy | No | No |
|  | EG38-J10UT | Pentax | Gastroscopy | No | No |
|  | EG-2990i | Pentax | Gastroscopy | No | No |
|  | G-EYE34-i10L | Pentax | ERCP | No | No |
| Storz (KARL STORZ SE & Co. KG, Tuttlingen, Germany) | | | | | |
| IMAGE1 S X-LINK | 13821 PKS | Storz | Gastroscopy | No | No |
