# Supplementary material for: Advancing artificial intelligence applicability in endoscopy through source-agnostic camera signal extraction from endoscopic images
Source: PLoS One. 2025 Jun 11;20(6):e0325987. doi: 10.1371/journal.pone.0325987 (PMC12157078; doi:10.1371/journal.pone.0325987)
Supplement: S2 Table — Description of the different small bowel and colon video capsules included in the EPIC dataset. EPIC: Endoscopic Processor Image Collection. (DOCX) [file pone.0325987.s002.docx]

**S2 Table:** **Image data from capsule endoscopy included in the EPIC dataset.** Description of the different small bowel and colon video capsules included in the EPIC dataset. EPIC: Endoscopic Processor Image Collection.

| **Manufacturer** | **Capsule** | **Diagnostic area** |
| --- | --- | --- |
| Medtronic | Pillcam SB2 | Small bowel |
|  | Pillcam SB3 | Small bowel |
|  | Pillcam Colon2 | Colon |
| Jinshan | OMOM HD Capsule | Small bowel |
