# Supplementary figures and images for: Advancing artificial intelligence applicability in endoscopy through source-agnostic camera signal extraction from endoscopic images
Source: PLoS One. 2025 Jun 11;20(6):e0325987. doi: 10.1371/journal.pone.0325987 (PMC12157078; doi:10.1371/journal.pone.0325987)

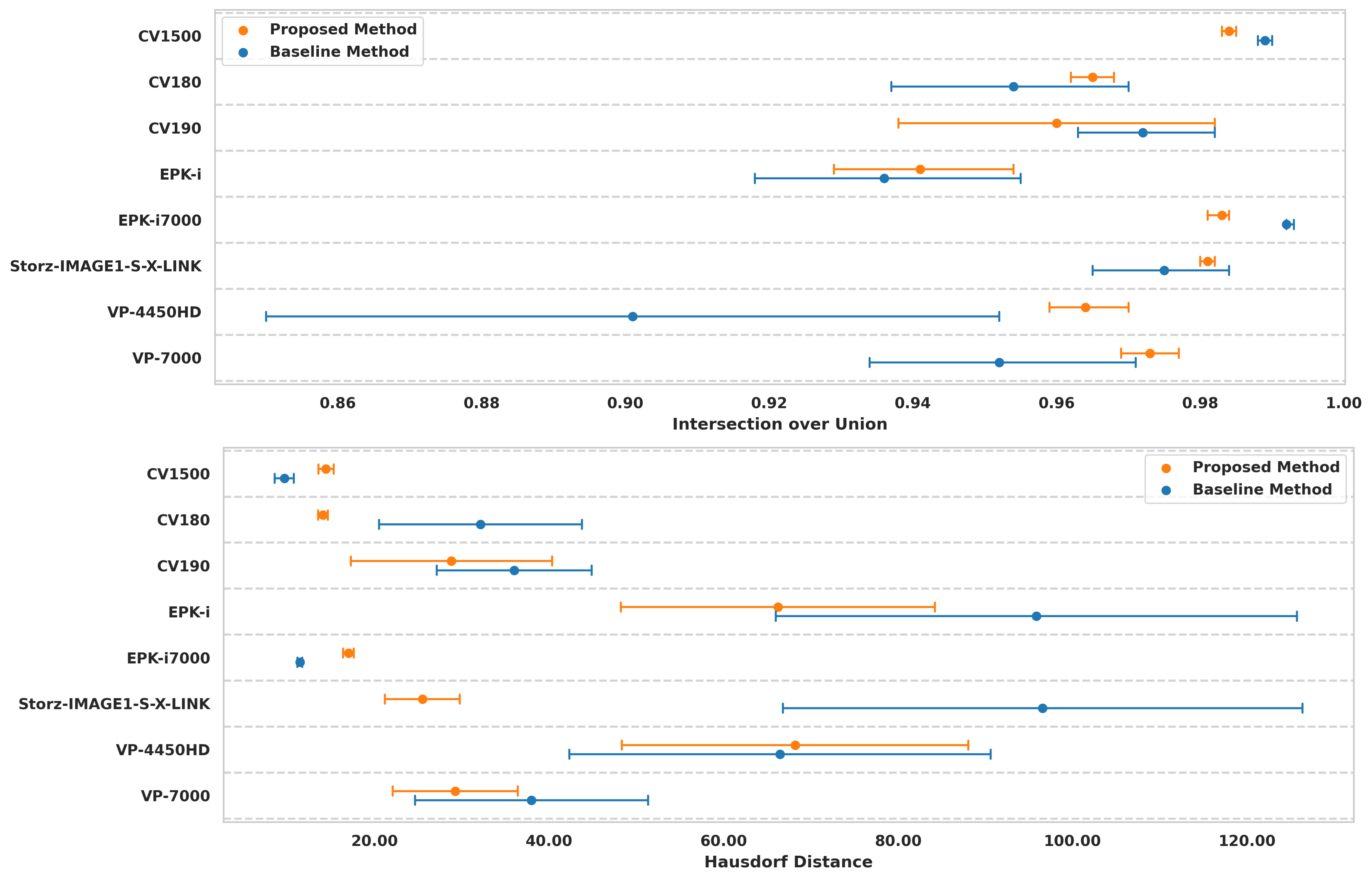

Supplement: S1 Fig — The subgroup analysis for each processor in terms of intersection over union and Hausdorff distance on the different processors included in the EPIC dataset is displayed. The mean value is indicated with a circle and lines depict 95% confidence intervals. (TIFF) [file pone.0325987.s003.tiff]
