# Supplementary material for: Advancing artificial intelligence applicability in endoscopy through source-agnostic camera signal extraction from endoscopic images
Source: PLoS One. 2025 Jun 11;20(6):e0325987. doi: 10.1371/journal.pone.0325987 (PMC12157078; doi:10.1371/journal.pone.0325987)
Supplement: S1 File — Description of the pipeline and training for the proposed method. (DOCX) [file pone.0325987.s004.docx]

# **Camera signal extraction pipeline and training of the camera image segmentation model.**

The proposed method takes any endoscopic image as input and predicts a binary mask that segments the camera signal in the input. To achieve this, the input is first scaled to 320x416 pixels and is input to an AI model that outputs a mask indicating the location of the camera signal. This mask is resized to the original image size and each pixel is compared to a threshold value to obtain a binary mask indicating the camera signal in the raw data. Using the predicted mask, a minimum dimension sub-image of the raw data containing the camera signal can be extracted, by taking the minimum and maximum row and column indices where the predicted mask has non-zero values.

We trained an AI model that segments the actual endoscopic image from irrelevant parts of an image such as the black padding and overlaid text. To perform image segmentation, a U-Net [1] was created using the segmentation-models-pytorch library [2] and an efficientnet b4 backbone [3] We chose the combination of binary cross-entropy with a dice loss, which is also known as combo loss [4] and AdamW [5] for optimization. Hyperparameter tuning for backbone and learning rate selection was performed using bayesian optimization. Backbones investigated were resnet-18 and resnet-34 and the efficientnet family (b1 - b4) and learning rates in the range 1e-5 to 1e-3. Training data followed an 80-20 split for train and validation. The model that performed best in a predefined budget of 50 epochs was selected. To reduce overfitting, augmentations are defined using the Albumentations [6] library, such as horizontal and vertical flipping, random brightness and contrast, random gamma. Additionally, two custom augmentations were defined to improve results on the dataset. The first one was the addition of random colored texts at different positions of the image, simulating written information that may appear in endoscopic images. The second augmentation, accounting for commonly displayed overlay boxes, such as warnings for low disk space in endoscopic images, displayed colored boxes on the image randomly, without affecting the mask.

## **Supplementary Bibliography:**

[1] Ronneberger, O., Fischer, P., & Brox, T. (2015). *U-Net: Convolutional Networks for Biomedical Image Segmentation* (arXiv:1505.04597). arXiv. https://doi.org/10.48550/arXiv.1505.04597

[2] Iakubovskii, P. (2019). *Segmentation Models Pytorch* [Python]. <https://github.com/qubvel/segmentation_models.pytorch>

[3] Tan, M., & Le, Q. V. (2020). *EfficientNet: Rethinking Model Scaling for Convolutional Neural Networks* (arXiv:1905.11946). arXiv. https://doi.org/10.48550/arXiv.1905.11946

[4] Taghanaki, S. A., Zheng, Y., Zhou, S. K., Georgescu, B., Sharma, P., Xu, D., Comaniciu, D., & Hamarneh, G. (2021). *Combo Loss: Handling Input and Output Imbalance in Multi-Organ Segmentation* (arXiv:1805.02798). arXiv. <https://doi.org/10.48550/arXiv.1805.02798>

[5] Loshchilov, I., & Hutter, F. (2019). *Decoupled Weight Decay Regularization* (arXiv:1711.05101). arXiv. <https://doi.org/10.48550/arXiv.1711.05101>

[6] Buslaev, A., Iglovikov, V. I., Khvedchenya, E., Parinov, A., Druzhinin, M., & Kalinin, A. A. (2020). Albumentations: Fast and Flexible Image Augmentations. *Information*, *11*(2), Article 2. https://doi.org/10.3390/info11020125
