# Supplementary material for: Advancing artificial intelligence applicability in endoscopy through source-agnostic camera signal extraction from endoscopic images
Source: PLoS One. 2025 Jun 11;20(6):e0325987. doi: 10.1371/journal.pone.0325987 (PMC12157078; doi:10.1371/journal.pone.0325987)
Supplement: Attachment — Submitted filename: Rebuttal-Letter.pdf [file pone.0325987.s006.pdf]

Rebuttal Letter for the Manuscript: Advancing artificial intelligence applicability in endoscopy through source-agnostic camera signal extraction from endoscopic images

Dear Professor Kazunori Nagasaka,

We would like to express our sincere gratitude for your thoughtful consideration and insightful comments on our manuscript. We have carefully addressed your suggestions and made the necessary revisions to enhance the clarity and impact of our work.

As recommended, we have expanded the discussion section to include a detailed explanation of how our proposed method can be integrated into both the development and application of AI in endoscopy. We have also highlighted how this integration can improve data preprocessing pipelines and AI model training for endoscopic image analysis. It now reads;

“We believe that our method can significantly enhance the generalizability of AI models trained for endoscopy. By integrating the proposed method into existing and future AI models, we achieve a streamlined process where raw endoscopic images are first processed through our method to extract only the camera signal, which contains all relevant information necessary for the AI model while minimizing irrelevant data, thereby standardizing the input images. This way, the images for the AI include all relevant information, a minimal amount of irrelevant information, and are as standardized as possible, independent of their source. This further contributes to standardizing endoscopic images, a process that has already been shown to significantly impact AI performance, for example, in esophageal cancer detection (43) and improved mucosal visualization in capsule endoscopy (44). Thus, the introduction of our method can greatly benefit the model’s ability to generalize data from any source without additional overhead. Furthermore, we believe that the proposed method can easily find successful application in pipelines for pre-processing endoscopic image data and AI model training. The fact that the image area to be cropped is selected as the minimum area rectangle containing all the mask pixels, together with the high performance of the AI, suggests that no loss of relevant information occurs from using the proposed method.”

We believe this expanded discussion better illustrates the potential impact of our method on clinical practice and future AI research in endoscopy. In addition, based on reviewer feedback, we have incorporated an analysis of execution time in the results section and extended the discussion to address potential limitations. This includes consideration of newer endoscopic hardware and the impact of endoscopic image content on our method’s performance. By incorporating these points, we aim to provide a more balanced perspective on the strengths and potential challenges of our approach.

Lastly, we have ensured that our manuscript aligns with the journal's required formatting style. This also included and updated version of the financial disclosure which now additionally states that “The funders had no role in study design, data collection and analysis, decision to publish, or preparation of the manuscript”.

We believe these revisions align with your expectations, and we greatly appreciate your support. Please find below our detailed, point-by-point response to each reviewer comment. I remain at your disposal for any further inquiries.

With kind regards, on behalf of all the authors,

Dr. Ioannis Kafetzis

Response to Reviewer 1 comments:

This manuscript proposed a camera signal extraction pipeline in endoscopy by using deep learning UNet model. Definitely the topic is interesting and important.

Response:

We thank you for your acknowledgment that the topic of the manuscript on a camera signal extraction pipeline in endoscopy using deep learning UNet models is important and interesting.

Comment 1:

The manuscript was poorly organised and written. English is poor, hard to follow. The quality of the figures are very low, even without labels at all.

Response:

We thank the reviewer for their feedback. We have thoroughly revised the paper to enhance expression and clarity. This included seeking help from a native English speaker to refine the language. Based on their feedback, we believe that the updated version is clearer. Additionally, we restructured the order in which points are presented in the methods section to facilitate a more intuitive reading experience. To ensure high-quality visuals, we generated all figures again at a resolution of 300 dpi to enhance clarity and detail. In Figure 5, we have added labels above each column of images to make the contents of each image clearer. With these revisions, we aim to improve the quality and readability of the manuscript.

Comment 2:

There exist lots of studies in AI for endoscopic image/video analysis as summarise in https://doi.org/10.1038/s41746-022-00733-3. Why do we need another one like the one in this manuscript, given that “the main contribution of this work is the development of an AI-based method that, given raw endoscopic image data as input, manages to extract, in a source agnostic way, the location of the endoscopic camera image from the raw data.”? – I do not see any new developments.

Response:

We thank the reviewer for their insightful comments and for highlighting that our work did not clearly articulate its main contributions. We have included the provided review and

characteristic references in our introduction to better contextualize our research. Additionally, we added more details about related methods attempting to extract camera signals from endoscopic images under the assumption of a circular shape, pointing out the need to extend these methos as camera signal in gastroscopies and colonoscopies do not typically appear in this form.

We believe this context makes it clear why we developed a source-agnostic method for extracting the endoscopic camera. Furthermore, we mention previous works attempting a similar approach to ours, but without extensive evaluation or making the model publicly available.

Comment 3:

Throughout the manuscript, authors talked about baseline models, but I am struggling to find what it is. It seemed to mention the paper by Yao (23) but that paper is about motion-based camera localization system in colonoscopy videos rather than simply segmenting images.

Response:

We appreciate the careful review and have revised the manuscript to address the feedback. Indeeed the paper of Yao (2023) is focused on motion-based camera localization in colonoscopy videos. Yet, in their section for data preparation they explicitly state the steps followed to extract the camera signal. We have made updated the description in our manuscript to pinpoint where the method is described in the original work. The passage now reads;

"To evaluate the efficiency of our proposed method, we compared our method with the currently used standard, which is described in (30), in the section 3.2 analyzing the data pre-processing pipeline."

Comment 4:

The results simply showed IoU and HD for the proposed method and "baseline method" – The analysis of effects of multi-centres and multi-camera devices is lacking.

Response:

We appreciate your comments and have revised address the issues you pointed out.

The reviewer is right to point out that the manuscript was missing a comparison of the impact that different hardware had. To address this, we added a subgroup analysis per endoscopic processor for IoU (Intersection over Union) and HD in the supplementary section, providing a more detailed comparison of the proposed method and the baseline method.

The reviewer also mentioned multi-camera devices. Unfortunately, the available endoscopes, we were only able to use standard monocular devices. Therefore, we did not have any information on multi-camera devices available in this study.

Comment 5:

In conclusion, the authors claimed that “This method can be used with any existing AI model to improve its ability to generalize to data from different sources, thus increasing transferability and sustainability.” – it is hardly convincing.

Response:

The reviewer is correct to point out that the conclusion is not convincing. To address this, we re-wrote the entire conclusion section and now reference specific applications of our method which demonstrate improved transferability and data sustainability. With these references, we believe that our claims are now better supported.

Here is the revised conclusion:

“In this work, we propose an AI-based method that effectively extracts camera signals from raw endoscopic recording data recording with different hardware and software options. This can enhance the standardization of images used as input to AI models, thereby increasing their transferability and generalizability across diverse clinical settings. This is crucial for maintaining consistent, high-quality data with reduced variability issues that can arise when training AI models on diverse datasets. Moreover, the proposed method's source agnosticism supports data sustainability by unifying diverse datasets into standardized format, simplifying their inclusion in AI training and evaluation pipelines.

Additionally, we generate a dataset, called EPIC, of standardized endoscopic images, attempting to highlight and collectively report the diversity that can be introduced by different endoscopic equipment.

By making both the proposed method and the EPIC dataset publicly available, we aspire to generate a collaborative environment where researchers can build upon these foundational resources to further advance their work in AI-driven endoscopic applications.”

Response to Reviewer 2 comments:

Comment 1:

The study assesses the AI-based strategy utilizing images from nine distinct processors and four capsule endoscopy equipment; yet endoscopic technology is perpetually advancing. Could the authors elaborate on the efficacy of their strategy when applied to newly designed or less frequently utilized endoscopic devices? What strategies might be employed to mitigate this constraint in further research?

Response:

The reviewer is right to point out that handling of data from newer devices has been noted in the discussion section. We acknowledge that endoscopic technology is rapidly advancing, and our AI-based strategy was tested with images from nine distinct processors and four capsule endoscopy equipment. However, we recognize that there are newer and less frequently utilized endoscopic devices that may pose challenges.

To address this constraint, we propose several strategies for future research:

Data Collection: We acknowledge the difficulty in obtaining relevant data from newer and higher-quality devices. To mitigate this, we encourage researchers to submit their datasets, which will help us update our model accordingly.

Model Generalization: Our AI-based strategy was validated on distinct and external datasets. Given its architecture, it is designed to maintain high performance even when applied to new data sources.

Public Release of Updated Models: We make the proposed method publicly available and welcome contributions from researchers who can provide additional datasets. Any updated versions of our model will be made available to ensure continued improvement and relevance.

"Endoscopy is a rapidly developing field, especially in terms of hardware. Furthermore, there are certainly sources of data that are rare, and thus obtaining relevant images can be harder. Considering this, the ability of the proposed method to perform on data from newer devices is significant. We are confident that the model can maintain high performance, as its evaluation was performed on two datasets that had no overlap with the training dataset. Furthermore, we make the model publicly available and welcome data submission from researcher to further develop and improve model performance. Any updated to the model will be also made available, acknowledging data contributions as well."

Comment 2:

Although the method attains elevated IoU and diminished Hausdorff distance on the EPIC dataset and public datasets, the study fails to address its efficacy in real-time clinical environments characterized by dynamic elements such as motion blur, lighting fluctuations, and aberrations. Could the authors address the possible limits of implementing this strategy in a clinical workflow and suggest measures to enhance its robustness?

Response:

We appreciate the reviewer's comments and understand the importance of evaluating the method's efficacy in real-time clinical environments. The following sections provide additional details on the implementation limits and measures to enhance robustness. The reviewer noted that the implementation in real-time was not explicitly mentioned, which is indeed a critical aspect for practical application. To address this concern, we have evaluated the mean time required for the model to process test data and translate it into frames per second (fps).

This analysis is included in the results section of our paper. In the worst-case scenario, the AI achieved a performance of 52 fps, which is more than satisfactory for incorporating into clinical workflows. The passage in the results section reads:

"Finally, the time required for the proposed method to extract the camera signal was investigated. In images from public datasets, the mean execution time per image was 0.011 seconds which is close to 90fps. For images on the EPIC datasets, which are in general of

higher resolution, the mean execution time per images was 0.018 seconds, or 52 fps. The results were obtained by using the method with an NVIDIA GeForce RTX 3080 Ti (NVIDIA Corporation, 2788 San Tomas Expressway, Santa Clara, CA 95051, USA). Both cases indicate that the model can achieve real-time performance."

The reviewer is also correct to highlight that low image quality data could impact model performance. We took this into account while developing the model, training with both high- and low-quality data. We now explicitly point this out in the corresponding methods section, which reads:
"The AI follows the UNet architecture (34) with an Efficient Net (35) as backbone. Training data for the model consisted of 1.765 manually annotated endoscopic images extracted from examination videos recorded between January 15th, 2019, and January 31st, 2022, in four different endoscopic centers. The training data included a range of image quality levels from clear, high-quality images to lower quality, blurry images. This diverse set of indicative images reflects the varied conditions encountered during endoscopy."

Finally, to address real time use, we added in the discussion section the following passage:
"Endoscopic data usually contain elements such as motion blur and artefacts, that could be disrupting to the AIs performance. To mitigate this problem, such data were also annotated and included in the training dataset. Furthermore, in case of video data, the cropping dimensions can be obtained via averaging of sequential frames, enabling removal of any outliers."

Comment 3:

The research contrasts the suggested method with only one published baseline. Could the authors elucidate the rationale for the exclusion of other existing AI-based segmentation algorithms from the comparative analysis? In what manner does this omission influence the study's capacity to thoroughly validate its superiority?

Response:

In the introduction, we now clearly state that our research contrasts the suggested method with only one publicly available baseline due to the specific focus on gastroenterology applications where circular endoscopic images are extracted, which is not a common scenario for other AI-based segmentation algorithms. This explains why certain existing methods were excluded from the comparison.

Additionally, in the introduction and conclusion section, we have emphasized that a recent paper used a UNET architecture for camera signal segmentation but did not provide any model or checkpoint, making it impossible to compare their results directly. Therefore, this method was also excluded from our comparative analysis.

Comment 4:

The article asserts that the proposed strategy enables AI models to generalize across various endoscope sources. Was this assertion corroborated by training and evaluating an

independent AI model on preprocessed photos from other sources? Otherwise, may the authors elaborate on the importance of performing such an evaluation?

Response:

The reviewer has pointed out that the assertion about the proposed strategy enabling AI models to generalize across various endoscope sources needs further clarification and validation. We appreciate this feedback and have made several updates to address these concerns.

To provide a more robust basis for our claim, we have added an explanation in the conclusion section regarding the usage of publicly available data for training or evaluation, as external validation. Furthermore, we argue that in one of our previous works, we trained an AI model using data from multiple endoscope sources. This allowed us to implement a polyp detection system across various clinics that use different hardware and software configurations. The passages read:

“I Inclusion of data from different sources has already proved beneficial in training AI models that find applications in clinical practice. As an example, diversity in data training allowed successful application of an AI in clinical routine in multiple different endoscopy centers in (45), achieving high performance despise the usage of different hardware used. Furthermore, studies have incorporated images from publicly available datasets as external validation, where standardization of images from different sources plays a central role (46).”

Comment 5:

The standardization of images from many sources may adversely affect nuanced image characteristics essential for specific AI-driven diagnostic applications. Can the authors address whether their strategy modifies clinically significant image details? How can this constraint be mitigated?

Response:

We acknowledge that standardization of images can sometimes lead to loss or distortion of clinically significant details. In the discussion section, we have clarified that our strategy does not involve any form of image standardization that could potentially compromise nuanced characteristics essential for specific AI-driven diagnostic applications.

We explicitly state in the discussion that there is no potential information loss due to the standardization process used in our method. We emphasize that our AI models are designed to achieve high accuracy and robustness through extensive training on diverse datasets. We explain that by directly using the area of the camera signal, we ensure minimal alteration to the original image characteristics, which is crucial for maintaining detail. The passage reads;

“The fact that the image area to be cropped is selected as the minimum area rectangle containing all the mask pixels, together with the high performance of the AI suggests that no loss of relevant information occurs from using the proposed method.”

Comment 6:

The authors must articulate a more compelling rationale for this study by detailing the imperative of creating a source-agnostic AI-driven segmentation technique for endoscopic pictures. The study effectively handles the issue of image variability from various endoscopic sources; nonetheless, it would benefit from a discussion on the role of standardization and preprocessing in enhancing AI-based medical image interpretation. Recent studies, including Chen et al. (2025), which assessed band selection strategies for improved visualization in esophageal cancer detection, and Wang et al. (2024), which illustrated the significance of hyperspectral imaging in augmenting mucosal visualization during capsule endoscopy, underscore the necessity of refining image processing techniques to elevate diagnostic precision. Referencing these works would enhance the rationale for the proposed method and situate its contribution within the wider progress in AI-enhanced endoscopic imaging:

1. Chen, Yen-Chun, et al. "Evaluation of Band Selection for Spectrum-Aided Visual Enhancer (SAVE) for Esophageal Cancer Detection." Journal of Cancer 16, no. 2 (2025): 470.

2. Wang, Yen-Po, et al. "Spectrum aided vision enhancer enhances mucosal visualization by hyperspectral imaging in capsule endoscopy." Scientific Reports 14, no. 1 (2024): 22243.

Response:

The reviewer is correct to highlight the importance of standardization in handling endoscopic data, and we appreciate the provided references which underscore the necessity of refining image processing techniques for enhanced diagnostic precision.

We have added these references to the conclusion section to provide a more comprehensive overview of the state-of-the-art in endoscopic image standardization. By incorporating these studies, we aim to strengthen our rationale for the proposed method and situate its contribution within the broader progress in AI-enhanced endoscopic imaging. This additional context will help readers better understand the importance of source-agnostic techniques and how they complement established methodologies like band selection and hyperspectral imaging. The passage reads:

“This way, the images for the AI include all relevant information, minimum amount of irrelevant information and are as standardized as possible, independent from their source. This further contributes to standardizing endoscopic images, a process that has been already shown to significantly impact AIs, for example in esophageal cancer detection (43) and improved mucosal visualization in capsule endoscopy (44). Thus, introduction of our method can greatly benefit the model’s ability to generalize data from any sources without any additional overhead.”
