# Supplementary material for: Advancing artificial intelligence applicability in endoscopy through source-agnostic camera signal extraction from endoscopic images
Source: PLoS One. 2025 Jun 11;20(6):e0325987. doi: 10.1371/journal.pone.0325987 (PMC12157078; doi:10.1371/journal.pone.0325987)
Supplement: Attachment — Submitted filename: REVISION2-Response-to-Reviewers.docx [file pone.0325987.s007.docx]

Dear Prof. Kazunori Nagasaka,

We would like to sincerely thank you for considering our manuscript for publication. We also apologize for the oversight regarding the missing figures in the revised manuscript. Please rest assured that we have carefully addressed all your comments, as well as those of the reviewers, in this revised version.

Below, we outline the actions taken in response to your specific concerns:

- **Comment 1:** Abstract Format: The abstract, particularly the last line, is improperly formatted. Please carefully proofread and format according to the journal's guidelines.

  **Response:** We apologize for the misalignment caused by applying text justification. This has now been corrected, and the abstract is properly aligned and formatted according to the journal's guidelines.
- **Comment 2:** Figures Missing: Figures 1 and 2 referenced in the text cannot be located. Ensure all figures referenced are clearly included and properly labeled. This was caused by a data size limit of the editorial manager.

**Response:** We sincerely apologize for this oversight. In the revised submission, all figures are now properly included, clearly labeled, and meet the journal’s high-quality standards, with a resolution of 300 dpi.

- **Comment 3:** Reference Accuracy: Reference 21 lacks essential publication information. Please verify and complete all references to comply with journal standards.

**Response:** We have updated Reference 21 by adding the missing publication details, including the proceedings in which the work was published, to ensure compliance with the journal’s referencing requirements.

- **Comment 4:** Technical Definition: The manuscript's definition of "Hausdorff distance (HD) as the maximum distance between predictions and the gold standard" is overly simplistic. Provide a more precise and technically accurate definition appropriate for a scholarly audience.

  **Response:** We recognize that the original definition was too simplistic. In the revised manuscript, we have included the formal mathematical definition of the Hausdorff distance and expanded the explanation to provide a more accurate and comprehensive description suitable for a scholarly audience.
- **Comment 5:** Gold Standard Generation: It remains unclear how the labels or gold standards for training were generated. Clarify your methodology for creating these standards to ensure reproducibility and transparency.

  **Response:** We have revised the Methods section to provide a detailed explanation of how the gold standard semantic segmentation masks were manually created for the camera signal, ensuring clarity and reproducibility of our methodology.
- **Comment 6:** Technical Contribution: The manuscript currently employs a UNet model without notable innovations or modifications. To enhance technical value, clearly state and elaborate on novel aspects or significant modifications of your approach compared to existing methodologies*.*

  **Response:** While we used a standard UNet model for segmenting the camera signal from raw endoscopic data, the primary contribution of our work lies in the application of an AI-based solution to standardize raw endoscopic images by extracting the camera signal. This approach addresses a critical gap in endoscopic research and is novel in its application. Additionally, we have made the trained model weights and methodology publicly available to support the broader research community. Our focus was on applying existing models in innovative ways to solve prevalent challenges in the field, rather than developing a new architecture. Yet, we took the change to highlight and clarify our goal and contributions better in the relevant parts of the introduction, conclusion and discussion sections.

We believe these revisions address all your concerns and have substantially improved the quality of the manuscript. A detailed response to the reviewer comments is also included below.

Once again, we are grateful for the valuable feedback from you and the reviewers, which has significantly enhanced the manuscript.

Dr. Ioannis Kafetzis

# Response to Reviewer #1

- **Comment:** The quality of the production is still quite low, say, the last line of the abstract is in improper format, there is no where to find Figure 1 and Figure 2, reference 21 has no publication information.

**Response:** We appreciate the reviewer’s careful review and apologize for the identified issues. Regarding the last line of the abstract, we resolved the formatting issue related to text justification. We have also ensured that Figures 1 and 2 are now properly referenced and visible in the manuscript. This was caused by a data size limit of the editorial manager. Finally, we have updated reference 21 with the necessary publication information.

- **Comment:** You cannot simply say "Hausdorff distance (HD) is the maximum distance between predictions and the gold standard."

**Response:** The reviewer is right to point out that the original description of the Hausdorff distance was overly simplistic. To address this, we have now provided a more precise and formal mathematical definition of the Hausdorff distance. Additionally, to make the concept clearer for a broader audience, we included an interpretation of the definition. The revised passage reads:
*The method was evaluated by means of intersection over union (IoU) and Hausdorff distance (HD). IoU is defined as the percentage overlapping with the gold standard. The HD, in the context of semantic segmentation, is defined as follows. Let* $m_{1}$ *and* $m_{2}$ *be two binary masks. The one-sided HD from* $m_{1}$ *to* $m_{2}$ *is defined as* $hd_{s}\left( m_{1},m_{2} \right)=\max_{x\in m_{1}} \min_{y\in m_{2}} \left\| x-y \right\|_{2}$*. This can be interpreted as the maximum distance from a point* $x\in m_{1}$ *to the closest point* $y\in m_{2}$*. The HD of* $m_{1}$ *and* $m_{2}$ *is defined as the maximum of the two one-sided HDs, that is* $HD\left( m_{1},m_{2} \right)=\max_{} (hd_{s}\left( m_{1},m_{2} \right), hd_{s}\left( m_{2},m_{1} \right)$*. Thus, the HD between the two masks can be seen as the greatest distance from a point in either mask to its nearest point in the other mask.*

- **Comment:** The design of the method in the manuscript is not clear how you generate the labels/gold standard for training.

**Response:** We appreciate the reviewer’s observation that the method for generating the gold standard was not sufficiently detailed. To clarify, we have expanded the relevant section in the Methods to explain that the gold standard semantic segmentation masks were manually created for the camera signal. The revised text reads:

*Gold standard binary semantic segmentation masks for each image were manually created to delineate the camera signal– where a value of 1 indicated pixel inclusion within the camera signal and 0 otherwise.*

- **Comment:** More importantly, the manuscript simply applied the UNet rather than developed some new techniques, which show very little technical contributions.

**Response:** While it is true that the paper utilizes a UNet architecture to segment the camera signal from raw endoscopic data, the primary contribution of the manuscript lies in applying AI-based methods to standardize raw endoscopic images. Our approach provides an applicable, practical solution for the research community, and the trained model weights, along with the method itself, are made publicly available to facilitate further research. The intention was not to propose a new architecture but to leverage existing, well-established methods to address a key challenge in the field. We believe this makes a meaningful contribution to the standardization of endoscopic data. We have updated the relevant parts of the abstract, introduction and discussion to highlight our aim and most importantly, the fact that the proposed method and model weights are publicly available.

# Response to Reviewer #2

We are thankful to the reviewer for the valuable insights they have provided. We are glad that they support publication of the work.
